# Generation of a restriction minus enteropathogenic *Escherichia coli *E2348/69 strain that is efficiently transformed with large, low copy plasmids

**DOI:** 10.1186/1471-2180-8-134

**Published:** 2008-08-05

**Authors:** Neil Hobson, Nancy L Price, Jordan D Ward, Tracy L Raivio

**Affiliations:** 1 Department of Biological Sciences, University of Alberta, Edmonton, AB, T6G 2E9, Canada; 2 Cancer Research UK, London Research Institute, Clare Hall Laboratories, Blanche Lane, South Mimms, EN6 3LD, UK

## Abstract

**Background:**

Many microbes possess restriction-modification systems that protect them from parasitic DNA molecules. Unfortunately, the presence of a restriction-modification system in a given microbe also hampers genetic analysis. Although plasmids can be successfully conjugated into the enteropathogenic *Escherichia coli *strain E2348/69 and optimized protocols for competent cell preparation have been developed, we found that a large, low copy (~15) bioluminescent reporter plasmid, pJW15, that we modified for use in EPEC, was exceedingly difficult to transform into E2348/69. We reasoned that a restriction-modification system could be responsible for the low transformation efficiency of E2348/69 and sought to identify and inactivate the responsible gene(s), with the goal of creating an easily transformable strain of EPEC that could complement existing protocols for genetic manipulation of this important pathogen.

**Results:**

Using bioinformatics, we identified genes in the unfinished enteropathogenic *Escherichia coli *(EPEC) strain E2348/69 genome whose predicted products bear homology to the HsdM methyltransferases, HsdS specificity subunits, and HsdR restriction endonucleases of type I restriction-modification systems. We constructed a strain carrying a deletion of the conserved enzymatic domain of the EPEC HsdR homologue, NH4, and showed that its transformation efficiency was up to four orders of magnitude higher than that of the parent strain. Further, the modification capacity of NH4 remained intact, since plasmids that were normally recalcitrant to transformation into E2348/69 could be transformed upon passage through NH4. NH4 was unaffected in virulence factor production, since bundle forming pilus (BFP) subunits and type III secreted (T3S) proteins were present at equivalent levels to those seen in E2348/69. Further, NH4 was indistinguishable from E2348/69 in tissue culture infection model assays of localized adherence and T3S.

**Conclusion:**

We have shown that EPEC strain E2348/69 utilizes a type I restriction-modification system to limit entry of new DNA. This restriction-modification system does not appear to be involved in virulence determinant expression or infection phenotypes. The *hsdR *mutant strain should prove useful in genetic analysis of the important diarrheal pathogen EPEC.

## Background

Restriction-modification systems are wide-spread in eubacteria and archaea and are thought to protect the host from bacteriophages, facilitate the gain of new genetic information, and allow for the maintenance of selfish genetic elements [1,2]. Type I restriction-modification systems were the first to be described and they are hetero-oligomeric enzymes consisting of a methyltransferase (HsdM), a specificity subunit (HsdS), and a restriction endonuclease (HsdR). The HsdR restriction endonuclease cleaves foreign DNA that has not been modified by the HsdM methyltransferase at a specific sequence recognized by the HsdS specificity subunit [1,2]. While this is an effective mechanism for protecting a microbe from newly encountered bacteriophages, it severely limits genetic analysis in many organisms, since new DNA is difficult to introduce. Indeed, most commonly used non-pathogenic commercial and laboratory strains contain deletions of *hsdR *homologues or entire type I restriction systems. We suspected the EPEC type strain E2348/69 might possess a restriction-modification system, since we had great difficulty in obtaining transformants that carried a large, low copy (~15 copies/cell) bioluminescent reporter plasmid, pJW15, that we modified for use in EPEC [3] and also since this strain cannot be infected with the *E. coli *generalized transducing phage P1.

EPEC is a leading cause of infantile diarrhea in the developing world [4]. Infection is thought to progress in three steps [5]. Initially, a type IV bundle forming pilus (BFP) mediates adherence to intestinal epithelial cells [6,7]. Following adhesion, a type III secretion system (T3SS) facilitates the transfer of translocator and effector proteins from the bacterial cytoplasm directly into the eukaryotic cytosol. One of these effectors, Tir, functions as a receptor in the eukaryotic cell membrane for the EPEC outer membrane protein intimin, fostering tight adherence between the microbe and the eukaryotic host cell [8]. In addition Tir, and other effectors, disrupt eukaryotic cellular processes, leading to microvillus effacement, tight junction disruptions, and changes in signal transduction that ultimately cause diarrhea [9]. Despite the health threat that EPEC poses, it remains relatively uncharacterized compared to its *E. coli *K-12 counterpart. One reason for this is likely due to the inability to efficiently introduce DNA through genetic techniques such as generalized transduction and transformation. Although a number of genetic techniques have been developed for use in EPEC based on conjugation [10,11] and optimized competent cell preparation [12], we wished to determine if a restriction-modification system might be responsible for the genetic intractability of EPEC strain E2348/69. If so, we reasoned that inactivation of such a restriction-modification system would render an additional useful tool for the EPEC research community.

## Results and discussion

### Identification of an *hsdR *homologue in the E2348/69 unfinished genome

**Figure 1:**
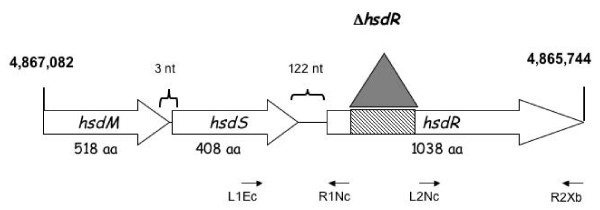
**A putative *hsdMSR *locus in E2348/69**. Large open arrows indicate *hsdM*, *hsdS*, and *hsdR *open reading frames. Numbers at left and right indicate positions in the unannotated E2348/69 genome. Primers used to construct a Δ*hsdR *allele are indicated at bottom of figure. The location of the *hsdR *deletion that removes the conserved helicase and ATP binding domains is indicated by a shaded box and an inverted triangle. aa, amino acid; nt, nucleotides.

New DNA cannot be introduced into E2348/69 by generalized transduction. Further, while we were modifying a bioluminescent gene reporter system for use in EPEC, we found that this strain was recalcitrant to transformation with the pJW15 plasmid (~10 kb) [3,13]. We hypothesized that E2348/69 might contain a restriction-modification system that could account for these observations. To determine if this was true, we used the sequences of some currently identified *hsdR *genes in GenBank to search for homology in the incomplete E2348/69 genome . We identified one predicted coding sequence (nucleotides 4862628–4865744 of the assembled sequence) that was 97% identical and 98% similar to the plasmid-borne *Eco*R124I HsdR protein over its entire length (1038 amino acids) [14]. The predicted E2348/69 HsdR protein also shared high homology with a number of other known or predicted HsdR homologues [15]. Upstream of the putative E2348/69 *hsdR *gene, we identified predicted *hsdM *(nucleotides 4867089–4868645 of the assembled sequence) and *hsdS *(nucleotides 4865866–4867092 of the assembled sequence) homologues (Figure 1). The predicted E2348/69 HsdM protein was 518 amino acids long and shared 98% identity and 99% similarity over its entire length with the *Eco*R124I HsdM protein and had high homology to other known or predicted HsdM proteins [15]. The putative HsdS protein was predicted to be 408 amino acids in length and shared limited homology with a number of predicted HsdS proteins. The predicted E2348/69 HsdS homologue shared the greatest similarity with a plasmid-borne HsdS from *Vibrio cholerae *(60% identity and 71% similarity over 401 of 408 amino acids). Since the HsdS protein confers sequence specificity to the restriction-modification enzyme complex, it is expected that HsdS homologues will share limited homology. As has been observed with other type I restriction-modification systems [1,2], the E2348/69 *hsdM *and *hsdS *genes appear to be in an operon, since they are separated by only 3 nucleotides, while the *hsdR *homologue is found 122 nucleotides downstream of *hsdS*, suggesting it may be expressed independently (Figure 1). Together, these observations imply that E2348/69 possesses a type I restriction-modification system that may limit the introduction of new DNA into this strain.

### An E2348/69 *hsdR *mutant exhibits elevated transformation efficiency and maintains HsdM activity

**Figure 2:**
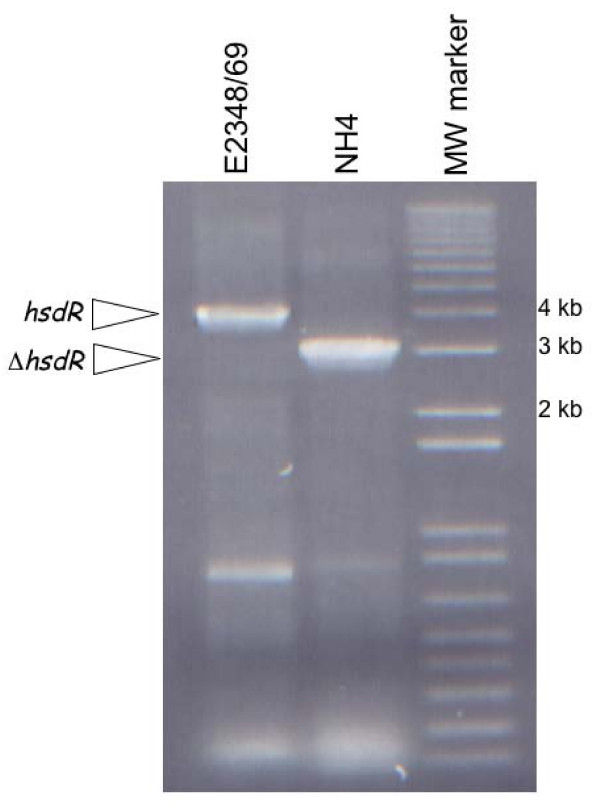
**EPEC strain NH4 carries a 1 kb deletion within the *hsdR *gene**. Agarose gel electrophoresis of products obtained after colony PCR of E2348/69 (left lane) and NH4 (right lane) using the hsdR-L1Ec and hsdR-R2Xb primers. Relevant molecular weight markers are indicated.

**Figure 3:**
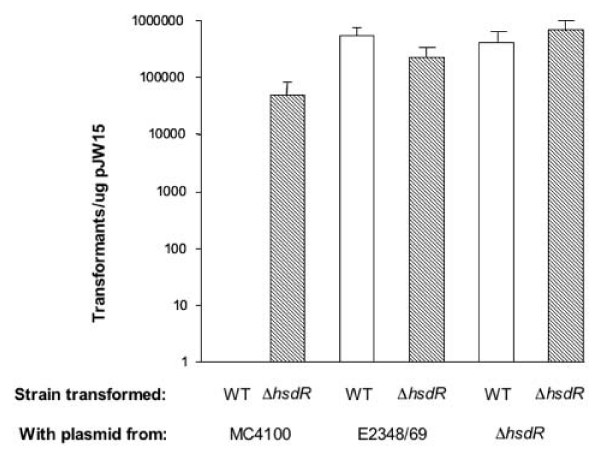
**The E2348/69 HsdMSR restriction-modification system is active**. Transformation efficiency testing of E2348/69 and NH4. Equal numbers of WT (E2348/69) and Δ*hsdR *mutant (NH4) bacteria were transformed with pJW15 plasmid and the number of transformants obtained was divided by the μg of plasmid used in the transformation to obtain transformation efficiency. E2348/69 and NH4 were transformed with pJW15 isolated from either MC4100, an *E. coli *K-12 strain, E2348/69, or NH4. Each experiment was performed three times and the data represent the mean and standard deviation.

We wished to determine if elimination of the putative *hsdR *homologue might render E2348/69 more competent for transformation. Thus, we engineered a construct that carried approximately 1.5 kb of DNA upstream and downstream from *hsdR *but that lacked approximately 323 codons of *hsdR *predicted to encode the ATP binding and helicase domains (Figure 1). These domains are highly conserved in all type I restriction endonucleases [1]. PCR analysis using primers that flanked *hsdR *revealed that the resultant strain, NH4, possessed an approximate 1 kb deletion, as predicted (Figure 2). To determine if NH4 could be more efficiently transformed than the parent strain E2348/69, we electroporated equal amounts of a large, promoterless *lux *reporter plasmid, pJW15 [3], isolated from our lab *E. coli *K-12 strain MC4100, into competent NH4 and E2348/69. We determined transformation efficiencies by dividing the total number of kanamycin resistant transformants detected by the amount of plasmid used in the transformation (Figure 1). No or very few E2348/69(pJW15) transformants were observed in multiple experiments (Figure 3, Table 1). This is consistent with our previous attempts to transform large plasmids into E2348/69 (often we perform multiple transformations at a time to isolate a single transformant). Conversely, we isolated almost 1 × 10^4 ^NH4(pJW15) transformants under the same conditions (Figure 3). We reliably witnessed increases in transformation efficiency with other large, low copy plasmids as well, including the *lux *reporter plasmid, pNLP10 (~10 kb, p15A origin, 10–12 copies/cell) [3,13], the 5 kb low copy cloning vector pACYC184 (5 kb, p15A origin, 10–12 copies/cell) [16], and the large cloning vector pLAFR1 (21.6 kb, RK2 origin, 5–7 copies per cell) [17]. Thus, NH4 can be transformed with large, low copy number plasmids like pJW15 with an efficiency that varies from three-fold to several orders of magnitude greater than that of E2348/69. We have previously had no difficulty transforming E2348/69 with small, high copy number plasmids like pUC19 or the pCA24-N plasmid used to construct the *E. coli *K-12 ASKA over-expression library [3,18].

**Table 1:** Transformation efficiencies of E2348/69 vs. E2348/69 Δ*hsdR*

**PLASMID**	**DESCRIPTION**	**SIZE**	**ORI**	**COPY#**	**TRANSFORMATION EFFICIENCIES^a^**	
					
					**E2348/69^b^**	**E2348/69^c^**
pJW15	lux reporter	~10 kb	p15A	10–12	2.5 × 10^-11^	8.3 × 10^-5^
pNLP10	lux reporter	~10 kb	pSC101	~5	0	5.4 × 10^-5^
pLAFR1	cosmid cloning vector	21.6	RK2	5–7	3.0 × 10^-9^	9.0 × 10^-7^

a. Transformation efficiencies are expressed as the (number of transformants/number of viable cells)/ug DNA. All experiments were repeated at least three times, and one representative experiment is shown.

b. Transformation efficiencies observed with plasmid isolated from *E. coli *K-12.

c. Transformation efficiencies observed with plasmid isolated from E2348/69Δ*hsdR*.

These data suggest that the introduced Δ*hsdR *allele does indeed make NH4 more competent for transformation with large, low copy number plasmids and argue that the HsdR endonuclease actively restricts incoming DNA in E2348/69. Further, this set of experiments shows that the *E. coli *K-12 strain MC4100 does not possess the E2348/69 HsdMSR restriction-modification system. Indeed, when the E2348/69 HsdR sequence was used in a BLAST search of the published *E. coli *K-12 genome , we detected only two proteins. A putative HsdR homologue shared only 23% identity over 181/1038 amino acids and the YejH protein of unknown function was 25% identical over 176/1038 amino acids. Both comparisons contained multiple, large gaps. Thus, *E. coli *K-12 does not contain the E2348/69 type I restriction-modification system identified here. As expected, this renders DNA isolated from *E. coli K-12 *(eg. MC4100) a poor substrate for transformation into EPEC (Figure 3).

Although type I restriction-modification systems consist of a hetero-oligomeric HsdMSR complex, it has been shown that a sub-complex consisting of HsdM and HsdS alone is competent for DNA modification [1]. Since the *hsdR *homologue is found downstream of the putative *hsdM *and *hsdS *genes in E2348/69 (Figure 1), we predicted that the Δ*hsdR *allele in NH4 would not disrupt the modification activities of the remaining HsdMS complex. We tested this hypothesis by determining the transformation efficiencies for E2348/69 and NH4, as described above, using pJW15 plasmid isolated from E2348/69 or NH4. In contrast to what we observed with plasmid isolated from MC4100, both E2348/69- and NH4-isolated pJW15 permitted the isolation of large numbers of both E2348/69(pJW15) and NH4(pJW15) transformants (Figure 3). These data suggest that DNA isolated from NH4 has been modified such that it escapes restriction by the EPEC HsdMSR complex upon transformation. To determine if other large, low copy plasmids might be similarly modified upon transformation into NH4, we transformed the *lux *reporter plasmid pNLP10 (10 kb, pSC101 origin, copy number ~5) [3], and the cloning vector pLAFR1 (21.6 kb, RK2 origin, copy number 5–7) [17] into NH4, reisolated the plasmids and used them to transform E2348/69 in parallel with the same plasmids isolated from an *E. coli *K-12 laboratory strain (Table 1). As previously observed, transformation efficiencies for the pJW15 plasmid increased several orders of magnitude when this plasmid was isolated from NH4 as compared to an *E. coli *K-12 laboratory strain (Table 1). Similarly, pNLP10 and pLAFR1 could both be transformed into E2348/69 at least two orders of magnitude better after they had been passaged through NH4 (Table 1), although transformation efficiencies were very low for the large 21.6 kb cosmid pLAFR-1. Accordingly, we conclude that both E2348/69 and NH4 contain active modification systems that permit plasmids isolated from these strains to be transformed into restriction-competent (E2348/69) hosts. Thus, disruption of the *hsdR *allele in NH4 leaves the modification activity of the predicted HsdMS complex intact.

### Mutation of *hsdR *does not render E2348/69 amenable to generalized transduction

In addition to being recalcitrant to transformation with large plasmids, E2348/69 is also resistant to infection with the *E. coli *generalized transducing phage P1. This is a serious drawback in genetic analysis of this organism, since the study of a given gene necessitates time consuming construction of mutant alleles and their recombination onto the E2348/69 chromosome by relatively cumbersome techniques. Creating strains carrying multiple mutant genes is even more tedious. Conversely, the movement of alleles between strain backgrounds by P1-mediated generalized transduction in *E. coli *K-12 can be accomplished in one day. In order to determine if the Δ*hsdR *mutation facilitated the movement of genetic material into E2348/69 by generalized transduction, we subjected NH4, E2348/69, and the *E. coli *K-12 strain MC4100 to P1 infection with phage lysates that had been grown on a strain carrying a *nadA*::Tn10 mutation. The *nadA*::Tn10 mutation confers tetracycline resistance as well as an inability to grow on unsupplemented minimal media. While we obtained hundreds of tetracycline resistant, minimal media deficient MC4100 *nadA*::Tn10 transductants, none were observed with E2348/69 or NH4. The same results were obtained with P1 lysates grown on strains carrying different mutant alleles that conferred various antibiotic resistant phenotypes. Thus, the HsdMSR restriction-modification system identified here is not responsible for the inability to infect E2348/69 with the P1 generalized transducing phage.

### Abrogation of *hsdR *does not affect virulence factor production in NH4

**Figure 4:**
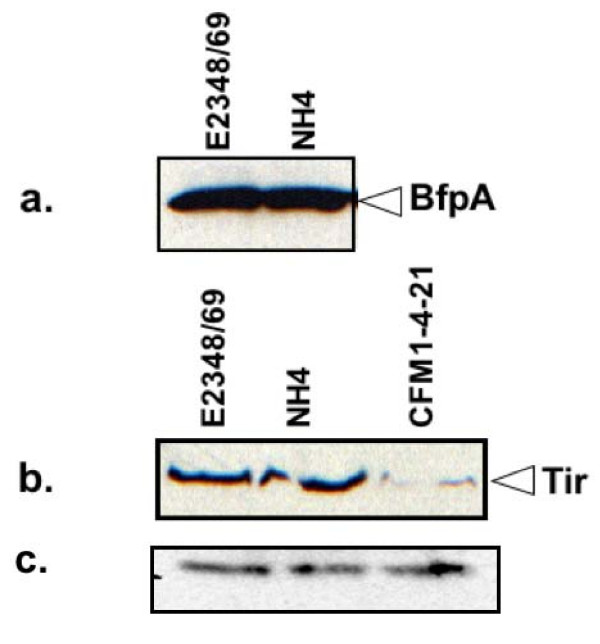
**A Δ*hsdR *mutant is unaffected in virulence factor production**. E2348/69 and NH4 were grown under conditions that stimulate virulence factor production and BfpA (a) levels were measured by western analysis of whole cell lysates. Secreted Tir (b) levels were measured by western analysis of SDS-PAGE gels after electrophoresis of precipitated supernatant samples. (c) Levels of a cross-reactive protein serve as a loading control. The experiment was performed twice and one experiment is shown.

**Figure 5:**
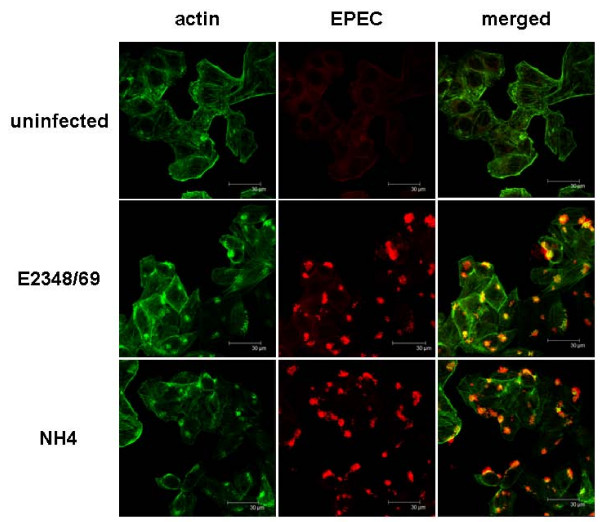
**NH4 is unaffected in T3S-mediated host cell intoxication**. E2348/69 and NH4 were used to infect HEp-2 tissue culture cells. Actin (green) and EPEC bacteria (red) were detected after staining with Alexa 488 phalloidin and rabbit anti-EPEC polyclonal anti-sera, followed by anti-rabbit antibody labeled with Cy3.

In order to use NH4 to facilitate molecular biological analysis of EPEC pathogenesis, it was necessary to demonstrate that this strain was unaffected in the regulated production of virulence determinants. Accordingly, we grew NH4, E2348/69, and relevant control strains under conditions previously shown to elevate virulence factor production, and assayed levels of two of the major virulence factors [19]. We found that the levels of the BFP subunit, BfpA, were unaffected in NH4 compared to E2348/69 (Figure 4a). BfpA is the major subunit of the BFP, which mediates initial adherence of EPEC to intestinal epithelial cells [6,7]. To confirm that adherence was not affected, we performed assays for localized adherence to tissue culture cells [20]. In two separate experiments with three replicates each, E2348/69 exhibited a localized adherence phenotype characterized by clusters of bacteria adhered to host cells on 72.3 +/- 2.8% of HEp-2 cells counted (1208 total), while NH4 displayed localized adherence to host cells on 69.2 +/- 3.3% of HEp-2 cells analyzed (1206 total). These numbers were not significantly different according to the students's *t *test (*P *= 0.5). Similarly, levels of Tir, a substrate for the T3SS, were comparable to those observed in E2348/69 (Figure 4b). In contrast, Tir secretion was dramatically down-regulated in the T3S mutant, CFM 14-2-1 (Figure 4b). To confirm that T3S was unaffected in NH4, we performed the fluorescent actin staining test (FAS) on cultures of HEp-2 cells infected with E2348/69 or NH4 (Figure 5). The FAS test measures actin rearrangements that occur upon T3S-mediated transfer of Tir to host cells, which leads to clusters of actin underneath of adhered EPEC bacteria [21]. We observed no discernible differences in actin staining after infection of HEp-2 cells with either E2348/69 or NH4 (Figure 5). In the case of E2348/69, out of 162 actin rearrangements, 152 were associated with the presence of E2348/69 bacteria (93.8%, Figure 5). For NH4, of 171 actin rearrangements, 164 NH4 microcolonies were observed (95.9%, Figure 5). Thus, the T3SS functions in strain NH4 the same as it does in the parent strain E2348/69. Together, these data demonstrate that virulence is unlikely to be affected by mutation of the E2348/69 *hsdR *homologue. Our findings agree with previous studies demonstrating that restriction endonuclease mutations have no effect on bacterial pathogenicity [22].

### The E2348/69 Δ*hsdR *mutant NH4 is a new tool for genetic analysis of EPEC infection

In this study we have identified an *hsdMSR *gene cluster in the E2348/69 genome and shown that mutation of the *hsdR *homologue produces a strain that can be transformed with large, low copy plasmids efficiently (Figure 3). Further, the production of the major EPEC virulence determinants in the Δ*hsdR *mutant, NH4, were unaffected (Figure 4) and we could discern no differences between E2348/69 and NH4 using tissue culture models of adherence and infection (Figure 5). These findings have important implications for the study of EPEC virulence. Although genetic techniques exist for conjugation of plasmids into E2348/69 [10,11], some plasmids, which are not amenable to conjugation, such as the pJW15 plasmid we used [3], will be much easier to work with using our newly developed Δ*hsdR *strain. This is a unique genetic tool that we expect will complement existing optimized techniques for preparing EPEC cells that are competent for transformation [12].

Although it should be possible to study pathogenesis directly in NH4 since our experiments indicate virulence determinant production is unaffected, we cannot say at this time whether NH4 may have diminished fitness relative to E2348/69 in vivo. It has been suggested that restriction-modification systems may provide an advantage to the bacterium in new environments where unfamiliar bacteriophages may be encountered [1,2]. Thus, it may be that the HsdMSR system identified here provides an advantage upon infection of the intestine. Even if this proved to be true, NH4 should still prove invaluable as a bridging strain. We have shown that the modification activity of the Hsd system identified here remains intact in the Δ*hsdR *NH4 mutant (Figure 3). Accordingly, exogenous DNA that is difficult to introduce into E2348/69 could first be introduced into NH4, where it would be modified, reisolated, and then moved into E2348/69. Indeed, we were able to use NH4 as an effective bridging strain for other large, low copy plasmids, including pLAFR1 and pNLP10. We are also hopeful that NH4 will improve the efficiency of other genetic techniques in EPEC that require the introduction of large, foreign DNA molecules, such as allelic exchange and transposon mutagenesis. We are currently testing these techniques in NH4. Thus, we hope that NH4 will be a useful tool to the EPEC research community.

## Conclusion

Genetic techniques that are routinely performed in laboratory strains of *E. coli*, such as generalized transduction and transformation, are impossible or orders of magnitude less efficient in unmodified pathogenic isolates. Because of this, genetic and molecular biological analysis of such microbes does not occur as rapidly as it does with "domesticated" strains. We modified a very low copy luminescent reporter plasmid for use in the EPEC type strain E2348/69, to monitor expression of genes of interest [3,13]. To our dismay, this plasmid, pJW15, was exceedingly difficult to transform into E2348/69, and we routinely had to do multiple transformations to acquire transformants. To determine if a restriction-modification system might be responsible for our troubles, we searched the E2348/69 genome for homologues of *hsd *restriction and/or modification enzymes [1]. In this paper, we report the identification of an operon encoding three genes with high homology to HsdM, HsdS, and HsdR proteins involved in DNA modification, restriction site specificity and DNA restriction. We engineered an E2348/69 strain lacking the conserved enzymatic domain of the HsdR protein, and demonstrated that this strain could be transformed orders of magnitude better than the wild-type strain with pJW15. The E2348/69Δ*hsdR *strain could also be transformed with other large, low copy plasmids bearing different replication origins, suggesting that this is a general attribute of this strain. Thus, the type I restriction-modification system encoded by these genes is active in E2348/69 and limits the acquisition of foreign DNA. The HsdMS enzyme complex remains functional for DNA modification in our Δ*hsdR *strain, since it can act as a bridging strain – pJW15, pLAFR1, or pNLP10 DNA that were passed through this strain could be transformed into the wild-type E2348/69 strain with ease. Other types of DNA modification can influence gene expression [23]. Thus, we examined virulence determinant expression in our Δ*hsdR *strain, since we desired to use this strain to study pathogenesis. We found no changes in growth or expression and function of two of the most important virulence determinants of EPEC; the type IV BFP which facilitates attachment to the intestine, and the T3SS, which mediates infection and intoxication of host cells. Thus, our strain will be useful for studying pathogenesis of EPEC, since it readily takes up large molecules of DNA and retains its key virulence properties – adherence to, and intoxication of, epithelial cells. No such strain currently exists, and so we regard this as a useful new tool for the EPEC research community.

## Methods

### Bacterial strains and plasmids

Bacterial strains and plasmids are listed in Table 2. Strains were grown on Luria-Bertani (LB) agar or liquid at 37°C in the presence of the appropriate antibiotic, 50 μg/ml kanamycin, 100 μg/ml ampicillin, 50 μg/ml streptomycin, or 25 μg/ml tetracycline (Sigma-Aldrich Co. Canada). For virulence determinant assays, E2348/69 derivatives were grown in Dulbecco's Modified Eagle's Medium (DMEM) as previously described [19,20].

**Table 2:** Strains and plasmids used in this study.

**Strain/Plasmid**	**Description**	**Source/Reference**
Strains		
E2348/69	Wild-type EPEC strain EPEC (O127:H6) isolated from an infant with gastroenteritis	[27]
MC4100	Wild-type E. coli K-12 lab strain F^- ^*araD139 Δ(argF-lac)U169 rpsL150*(Str^R^) *relA1 flbB5301 deoC1 ptsF25 rbsR*	[28]
TOP10 cells	Commercially available competent cells for cloning F^- ^*mcrA Δ(mrr-hsdRMS-mcfBC) φ80lacZΔM15 ΔlacX74 recA1 araΔ139 Δ(ara-leu) 7697 galU galK rpsL endA1 nupG *(Str^R^)	Invitrogen Canada Inc.
TAM1 λpir	Commercially available competent cells of *E. coli *λlysogen that provide all trans acting and mobilization factors required for the replication and mobilization of λPi dependent plasmids *mcrA Δ(mrr-hsdRMS-mcfBC) *φ80*lacZ*ΔM15 *ΔlacX74 recA1 araΔ139 Δ(ara-leu) 7697 galU galK rpsL endA1 nupG λpir*	Active Motif
SM10 λpir	*E. coli *SM10 λlysogen that provide all trans acting and mobilization factors required for the replication and mobilization of λPi dependent plasmids	[29]
NH1	TOP10 (pUC19 Δ*hsdR*)	This study
NH3	SM10 λ*pir *(pCVD442 Δ*hsdR*)	This study
NH4	E2348/69 Δ*hsdR*	This study
LP69	MC4100 *nadA::Tn10*	Lab stock
		
Plasmids		
pACYC184	5 kb low copy number cloning vector	[16]
pUC19	Cloning vector	Invitrogen Canada Inc.
pUC19 Δ*hsdR*	pUC19 carrying Δ*hsdR *construct	This study
pCVD442	Cloning vector requiring λ Pi protein to replicate, carries *sacB *for negative selection (Amp^R^)	[10]
pCVD442 Δ*hsdR*	pCVD442 carrying Δ*hsd*R construct	This study
pJW15	Broad host range promoterless *lux *reporter plasmid	[3,13]
pLAFR1	Low copy number, broad host range plasmid	[17]
pNLP10	Low copy number lux reporter plasmid, pSC101 origin	[3,13]

a. Transformation efficiencies are expressed as the (number of transformants/number of viable cells)/ug DNA. All experiments were repeated at least three times, and one representative experiment is shown. b. Transformation efficiencies observed with plasmid isolated from *E. coli *K-12. c. Transformation efficiencies observed with plasmid isolated from E2348/69Δ*hsdR*.

### Construction of a E2348/69 *hsdR *mutant

DNA fragments encoding the amino and carboxyl terminal portions of the E2348/69 HsdR homologue were amplified from the E2348/69 chromosome using the restriction site-tagged primer pairs HsdR-L1Ec (5'-GGGAATTCGTTAGTCTACCAATGGGCGAC-3', *Eco*RI tag) and HsdR-R1Nc (5'-CGCCATGGTGCCACTCGCTGTCATTAAAC-3', *Nco*I tag) or HsdR-L2Nc (5'-CGCCATGGATTTGATGAATGCCACCGCAG-3', *Nco*I tag) and HsdR-R2Xb (5'-GGTCTAGAGATTGCGGGTTTAACGGACTG-3', *Xba*I tag), respectively (restriction sites underlined). The PCR program used cycled the reaction at 95°C for 1 minute, 48°C for 1 minute, and 72°C for 2 minutes, 35 times and finished with a 72°C, 4 minute extension followed by a hold at 4°C. Using standard cloning procedures, equal amounts of the two purified PCR fragments were digested with *Nco*I and ligated to form a product encoding an N-terminal deletion of the predicted conserved helicase and ATP binding domains of HsdR. This fragment was digested with *Xba*I and *Eco*RI (Invitrogen Canada Inc.) and cloned into the same sites in pUC19. In order to recombine the Δ*hsdR *allele onto the E2348/69 chromosome, the pUC19:Δ*hsdR *construct was digested with *Eco*RI and the recessed ends were filled in using Klenow fragment. This product was digested with *Xba*I and the resulting Δ*hsdR *fragment was cloned into the *Xba*I and *Sma*I sites of the gene replacement vector pCVD442 [10]. This construct was conjugated into E2348/69 and double recombinants that contained the Δ*hsdR *allele were sequentially selected for by antibiotic resistance and sucrose sensitivity as previously described [24]. The resulting colonies were screened for the presence of the Δ*hsdR *allele via PCR using the primers HsdR-L1Ec and HsdR-R2Xb. One positive isolate was named NH4.

### Transformation efficiency tests

Electroporation competent cells were prepared using standard techniques from equal numbers of E2348/69 and NH4 by normalizing culture volumes according to absorbance measured at 600 nm. Plasmid samples were prepared with the GenElute Plasmid Miniprep Kit (Sigma-Aldrich) and DNA concentrations determined by measuring the absorbance at 260 nm. Equal volumes of competent EPEC and NH4 cells were transformed with 1 uL of the same plasmid preparation using a BioRad MicroPulser electroporator set on the bacterial setting and 2 mm gap electroporation cuvettes. The transformed cells were serially diluted and 100 uL of each dilution were plated on LB plates containing the appropriate antibiotic and in some cases to LB plates lacking antibiotics to ascertain the number of viable cells. The transformation efficiency was calculated as the total number of transformants divided by the amount of plasmid used in the transformation (μg) or by dividing the total number of transformants obtained by the number of viable cells and then dividing this number by the amount of DNA used in the transformation. All transformation efficiencies were determined at least three times. Although overall numbers varied depending on the plasmid preparation or batch of competent cells used, the trends within experiments remained the same over multiple repetitions. In Table 1, one representative experiment is shown.

### Assays of virulence determinant production and infection phenotypes

BfpA, and Tir levels were measured as previously described [19,20]. BfpA and Tir were assessed by western blot analysis (α-BfpA courtesy of M. Donnenberg, U. Maryland, α-Tir courtesy of B. Finlay, UBC). As a loading control, a cross-reactive protein was included in Figure 2c. Assays for localized adherence were performed as previously described [20,25]. The FAS assay was adapted from Knutton *et al. *[21] and DeVinney *et al. *[25]. Briefly, HEp-2 cells were seeded on coverslips in a 24 well plate at a concentration of 2 × 10^5 ^cells/mL and grown overnight at 37°C/5% CO_2_. Bacterial strains were inoculated in LB and grown statically overnight at 37°C/5% CO_2_. The HEp-2 monolayers were infected with 5 μL of static bacterial culture for 2.5 h. Specimens were washed thoroughly (4 times) with phosphate buffered saline (PBS) and fixed with 2.5% paraformaldehyde for 10 min at 37°C. Samples were washed with PBS (4 times) then permeabilized with PBS/0.1% Triton X-100/10% Fetal Bovine Serum (FBS, Invitrogen) for 30 min at 37°C. Antisera for EPEC (1:300, R. DeVinney U. Calgary) was added to the specimens for 30 min at 37°C. After washing again with PBS (4 times), specimens were stained with anti-rabbit-Cy3 (1:400, R. DeVinney. U. Calgary) and Alexa 488 phalloidin (1:400, Molecular Probes) for 30 min at room temperature in the dark. Samples were washed with PBS once more and then mounted for viewing. Confocal images were obtained using a Leica fluorescence microscope (BioSci Microscopy Unit) at 60× objective.

### Generalized transduction

Generalized transduction was performed using routine procedures as previously described [26].

## Authors' contributions

NH carried out the experiments and drafted portions of the manuscript. NLP performed experiments and wrote portions of the manuscript. JDW conceived of the study and identified the *hsd *genes in the EPEC genome. TLR participated in the design of the experiments, obtained funding, supervised NH, NLP, and JDW, and wrote portions of the manuscript. All authors have read and approved the final manuscript.
